# Sex differences in the impact of multimorbidity on long-term mortality for patients with colorectal cancer: a population registry-based cohort study

**DOI:** 10.1093/pubmed/fdaf012

**Published:** 2025-02-05

**Authors:** Shu Kay Ng, Peter Baade, Gary Wittert, Alfred K Lam, Ping Zhang, Saras Henderson, Belinda Goodwin, Joanne F Aitken

**Affiliations:** School of Medicine and Dentistry, Griffith University, Gold Coast, QLD 4222, Australia; Cancer Council Queensland, Fortitude Valley, QLD 4006, Australia; Centre for Data Science, Queensland University of Technology, Brisbane, QLD 4000, Australia; School of Public Health, University of Queensland, Herston, QLD 4006, Australia; Freemasons Centre for Male Health and Wellbeing, South Australian Health and Medical Research Institute and University of Adelaide, Adelaide, SA 5000, Australia; School of Medicine and Dentistry, Griffith University, Gold Coast, QLD 4222, Australia; School of Medicine and Dentistry, Griffith University, Gold Coast, QLD 4222, Australia; School of Nursing and Midwifery, Griffith University, Gold Coast, QLD 4215, Australia; Cancer Council Queensland, Fortitude Valley, QLD 4006, Australia; Centre for Health Research, University of Southern Queensland, Springfield Central, QLD 4300, Australia; Melbourne School of Population and Global Health, University of Melbourne, Carlton, VIC 3053, Australia; Cancer Council Queensland, Fortitude Valley, QLD 4006, Australia

**Keywords:** colorectal cancer, long-term mortality, multimorbidity, registry-based cohort study, sex difference

## Abstract

**Background:**

Women have better survival than men patients with colorectal cancer (CRC), but the extent to which this is due to multimorbidity is unclear.

**Methods:**

A population-based study of 1843 patients diagnosed with CRC in Australia. Data included patient’s demographics, multimorbidity, tumour histology, cancer stage, and treatment. We estimated the risks of all-cause mortality and cause-specific mortality due to cancer or non-cancer causes.

**Results:**

Men had lower survival than women (*P* ≤ 0.010) amongst those diagnosed at Stages I-III (15-year survival: 56.0% vs 68.0%, 48.5% vs 60.7%, 34.8% vs 47.5%, respectively), excepting Stage IV (14.4% vs 12.6%; *P* = 0.18). Married men exhibit better survival than those who were never married (*P* = 0.006). Heart attacks (9.9% vs 4.3%, *P* < 0.001) and emphysema (4.8% vs 2.1%, *P* = 0.004) were more prevalent in men than women. Comorbid stroke and high cholesterol (adjusted hazard ratio, AHR = 2.22, 95% confidence interval, CI = 1.17–4.21, *P* = 0.014) and leukaemia (AHR = 6.36, 95% CI = 3.08–13.1, *P* < 0.001) increased the risk of cancer death for men only. For women, diabetes increased the risk of all-cause death (AHR = 1.38, 95% CI = 1.02–1.86, *P* = 0.039) and high blood pressure increased the risk of death due to non-cancer causes (AHR = 2.00, 95% CI = 1.36–2.94, *P* < 0.001).

**Conclusion:**

Separate models of CRC care are needed for men and women with consideration of multimorbidity and social factors.

## Introduction

Cancer is one of the most pressing public health problems worldwide. In Australia, colorectal cancer (CRC) is the second and third most diagnosed cancer for women and men, respectively. It accounts for 11% of all cancer deaths and is ranked the third highest cause of cancer-related mortality.^1^ The impact of multimorbidity (chronic non-communicable diseases (NCDs) distinct from the principal cancer diagnosis)^2^ pose a major challenge in delivering optimal treatment and excess public health burden on the healthcare system.^3–5^ Cancer patients with multimorbidity were underrepresented in clinical trials, resulting in a lack of representativeness amongst participants in intervention trials.^6^ Clinical treatment guidelines for CRC recommend multimorbidity status should be carefully reviewed in multidisciplinary team (MDT) meetings.^7^^,^^8^ However, the lack of published evidence on prognosis for cancer patients with specific multimorbidity is a barrier to reaching a clear treatment plan.^9^^,^^10^ Thus, surgery for these patients is often delayed, treatments are generally more variable, adverse drug reactions are more common, and multimorbidity is inadequately addressed and health-related quality of life is poor.^1^^,^^5^^,^^11^

Many patients die from complications of non-cancer chronic NCDs rather than their cancer. Previous studies on the short-term survival of CRC patients have shown that patients with multimorbidity had two times or higher mortality risk compared to patients without multimorbidity.^9^^,^^12^ These non-cancer comorbid NCDs vary in severity and disease combinations and affect patients in an unpredictable manner.^13–15^ But studies on long-term mortality from the time of cancer diagnosis are relatively sparse.

Despite there being minimal sex differences in screening uptake, routes to diagnosis, cancer staging at diagnosis, and effectiveness of treatment,^16^ women have better survival after a diagnosis of CRC compared to men.^17^^,^^18^ Population-based studies of CRC patients revealed sex differences in treatment allocations and survival advantage of women in young and middle-aged patients with localized disease.^19^^,^^20^ However, there is limited information as to the prognostic factors (especially, multimorbidity) associated with poorer CRC survival amongst men.

This study aims to use data from a population registry-based cohort study to investigate sex differences in 15-year long-term mortality and associated prognostic factors including multimorbidity, demographic, socioeconomic, and geographical factors for men and women with CRC. Specifically, we wished to determine the effects of prognostic factors on mortality across different populations, including all-cause mortality as well as deaths attributed to cancer or non-cancer causes separately. The findings will contribute to developing public health interventions to improve the prognosis of CRC patients, cancer survivor’s health and quality of life.

## Methods

### Data source and study design

The study cohort is from a population registry-based longitudinal study of 1966 patients diagnosed with primary CRC (the International Classification of Diseases 10th Revision, ICD-10 codes C18-C20) in Queensland in 2003–2004 conducted by the Cancer Council Queensland (CCQ). Eligible patients aged 20–80 years were identified from the Queensland Cancer Registry (QCR). The treating doctors of eligible patients were approached in writing for permission to contact their patients regarding the study.^21^ The patients with doctor granted consent were mailed an information sheet and a consent form. The response rate amongst this cohort was 61.8% amongst 3182 eligible patients invited to participate.^21^ There was no difference in the distributions by sex of the study cohort and the total group of eligible patients.^3^ Apart from the information obtained through the QCR, patient-level data were acquired from computer-assisted telephone interviews (Supplementary Table S1). Data linkage with the QCR provided information on deaths up to 31 December 2019. Key variables include data on pre-existing chronic NCDs, cancer stage and site at diagnosis, treatment, community-level factors (geographical remoteness, socioeconomic status in the patient’s location of residence), and 15-year survival outcomes (including cause of death categorized into cancer and non-cancer causes). We adhered to Strengthening the Reporting of Observational studies in Epidemiology (STROBE) reporting guidelines.

### Pre-existing chronic non-communicable diseases and other risk factors

Participants were asked to identify on a checklist (Supplementary Table S1) any chronic NCDs they had been diagnosed with. Participants with a body mass index >30 were classified as obese according to World Health Organization recommendations. These diagnosed conditions were considered in the analysis and were referred to as multimorbidity because these conditions, except heart attacks, angina pectoris and stroke, reflected pervasive chronic diseases that are likely to be comorbid with the CRC diagnosis.^3^

The cancer stage at diagnosis was determined according to the TNM system using information extracted from pathology forms.^22^^,^^23^ Cancer sites were classified as colon or rectum. Treatments received were obtained through patient interviews. The response was grouped into three categories: Surgical only, Surgical plus adjuvant (including chemotherapy or radiotherapy), and Others (including awaiting treatment or no treatment). Location of residence was grouped into three categories: Metropolitan, Inner-regional, and Outer-regional/Remote areas.^3^ Based on the location, the Index of Relative Socioeconomic Advantage and Disadvantage (IRSAD) was assigned to reflect the average community-level socioeconomic status (SES) for each participant. The quintile of IRSAD represents the increasing socioeconomic advantage (Quintile 1, most disadvantaged).

### Outcome variables

The mortality status of each participant on 31 December 2019 was obtained from the QCR by linking with the data of the State Government Department of Births, Deaths, and Marriages. The data included date and cause of death, which was categorized into cancer and non-cancer causes. The dataset also contained specific causes of death for cancer deaths (defined by ICD-10 codes C00-C96) whilst non-cancer deaths were grouped into one category. Time to death was taken to be the difference between dates of diagnosis and death. For participants who were still alive on 31 December 2019, their times since diagnosis were calculated up to that date and were censored.

### Statistical analysis

Continuous variables were reported as means or medians with range, whereas categorical variables were expressed as frequency and percentages. The independent-sample t-test (or Mann–Whitney test) was used to compare means (or medians) between sexes for continuous variables. The chi-square test was used to explore sex differences in categorical variables. Survival analyses were performed using a flexible parametric survival model on the log cumulative-hazard scale, via the ‘stpm2’ command in Stata SE-16 (StataCorp, College Station, TX).^24^ The model uses restricted cubic spline functions to capture complex baseline hazard functions and time-dependent (non-proportional) effects. The proportional hazards assumption [constant hazard ratio (HR) over time] was tested using the Schoenfeld method. If this proportionality assumption was violated, a cubic spline to model the interaction of the non-proportional factor with time was added to handle the change of HR over time. The number of degrees of freedom for the baseline spline function and time-dependent effects was selected based on the Bayesian information criterion (BIC), which is amongst the most used method for model selection.^25^

Risks of all-cause mortality (overall survival) were estimated first. Population-averaged survival and adjusted HRs for any time-varying effects were estimated using the ‘prediction’ command in ‘stpm2’. Cause-specific cumulative incidence functions (CIFs), the ‘crude’ probabilities of cancer and non-cancer deaths, were then estimated using ‘stpm2cif’ in Stata.^26^ In estimating CIFs, each patient’s categorical factor was characterized by the level with the highest frequency. This is better than estimating probabilities at the mean value of categorical factors (which do not have meaningful interpretation).

## Results

We considered 1843 participants (93.7% of the original cohort) with information on the cancer stage at diagnosis. Table 1 shows the cohort characteristics and self-reported prevalence of multimorbidity by sex. Women patients were younger at diagnosis (mean age: 63.8 vs. 65.2 years, *P* = 0.005). The distribution of cancer stage at diagnosis was similar between men and women. However, compared to men, a significantly higher proportion of women had colon cancer (76.0% vs. 66.4%, *P* < 0.001) and treatment comprising surgery alone (58.3% vs. 51.1%, *P* = 0.003). Amongst the participants, a higher proportion of women were widowed or divorced compared to men (31.3% vs. 15.0%, *P* < 0.001). Educational level, private health insurance status, geographical features (residential remoteness and community-level SES) were not significantly different between sexes (*P* > 0.05). Regarding multimorbidity, women had a higher prevalence of asthma, osteoporosis, osteoarthritis, migraine, and depression (Table 1, individual *P* < 0.005), whilst men had a higher prevalence of heart attacks and emphysema (individual *P* < 0.005). Overall, 1613 (87.5%) patients had at least one comorbid NCD (women: 86.9%, men: 87.9%, no significant sex difference). However, amongst patients with comorbid NCD, the median number of NCDs in women was higher (median: 3 vs. 2, *P* = 0.005; range: 1–14 vs. 1–10).

**Table 1:** Sex differences in cohort characteristics and prevalence of multimorbidity.

Characteristic	Overall (n = 1843)	Women (n = 748)	Men (n = 1095)	*P*-value
Age at diagnosis	64.6 (21.1–81.0)	63.8 (22.8–81.0)	65.2 (21.1–81.0)	0.005
Stage at diagnosis I II III IV	456 (24.7%) 649 (35.2%) 553 (30.0%) 185 (10.0%)	191 (25.5%) 263 (35.2%) 227 (30.4%) 67 (9.0%)	265 (24.2%) 386 (35.3%) 326 (29.8%) 118 (10.8%)	0.61
Site Colon Rectum missing	1180 (70.3%) 499 (29.7%) 164	516 (76.0%) 163 (24.0%) 69	664 (66.4%) 336 (33.6%) 95	<0.001
Treatment type Other treatment Surgery alone Surgery + adjuvant therapy	63 (3.4%) 996 (54.0%) 784 (42.5%)	17 (2.3%) 436 (58.3%) 295 (39.4%)	46 (4.2%) 560 (51.1%) 489 (44.7%)	0.003
Marital status Never married Married/defacto Widowed/Divorced	77 (4.2%) 1368 (74.2%) 398 (21.6%)	34 (4.6%) 480 (64.2%) 234 (31.3%)	43 (3.9%) 888 (81.1%) 164 (15.0%)	<0.001
Education level Less than 8 years 8–12 or college University	254 (13.8%) 1342 (72.8%) 247 (13.4%)	106 (14.2%) 553 (73.9%) 89 (11.9%)	148 (13.5%) 789 (72.1%) 158 (14.4%)	0.29
Private health insurance No Yes	746 (40.5%) 1097 (59.5%)	305 (40.8%) 443 (59.2%)	441 (40.3%) 654 (59.7%)	0.83
Geographical remoteness Metropolitan Inner regional Outer regional/Remote	904 (49.1%) 597 (32.4%) 342 (18.6%)	363 (48.5%) 236 (31.6%) 149 (19.9%)	541 (49.4%) 361 (33.0%) 193 (17.6%)	0.45
SES Quintile 1 (lowest) 2 3 4 5 (highest) missing	308 (16.8%) 307 (16.7%) 346 (18.9%) 610 (33.3%) 263 (14.3%) 9	121 (16.2%) 112 (15.0%) 143 (19.2%) 258 (34.6%) 111 (14.9%) 3	187 (17.2%) 195 (17.9%) 203 (18.6%) 352 (32.3%) 152 (14.0%) 6	0.48
Multimorbidity				
Heart attacks	140 (7.6%)	32 (4.3%)	**108 (9.9%)**	<0.001
Angina Pectoris	182 (9.9%)	60 (8.0%)	122 (11.1%)	0.03
High blood pressure	747 (40.5%)	296 (39.6%)	451 (41.2%)	0.49
High cholesterol	530 (28.8%)	230 (30.8%)	300 (27.4%)	0.12
Other heart condition	292 (15.8%)	102 (13.6%)	190 (17.4%)	0.03
Stroke	74 (4.0%)	26 (3.5%)	48 (4.4%)	0.33
Asthma	233 (12.6%)	**115 (15.4%)**	118 (10.8%)	0.004
Chronic bronchitis	114 (6.2%)	54 (7.2%)	60 (5.5%)	0.13
Emphysema of lungs	68 (3.7%)	16 (2.1%)	**52 (4.8%)**	0.004
Osteoporosis	108 (5.9%)	**91 (12.2%)**	17 (1.6%)	<0.001
Osteoarthritis	301 (16.3%)	**155 (20.7%)**	146 (13.3%)	<0.001
Rheumatoid arthritis	102 (5.5%)	54 (7.2%)	48 (4.4%)	0.009
Leukaemia	15 (0.8%)	7 (0.9%)	8 (0.7%)	0.63
Diabetes	230 (12.5%)	81 (10.8%)	149 (13.6%)	0.08
Stomach or duodenal ulcer	229 (12.4%)	75 (10.0%)	154 (14.1%)	0.01
Migraine headaches	245 (13.3%)	**164 (21.9%)**	81 (7.4%)	<0.001
Depression	254 (13.8%)	**126 (16.8%)**	128 (11.7%)	0.002
Any previous cancer	362 (19.6%)	145 (19.4%)	217 (19.8%)	0.82
Obese (BMI > 30)	476 (25.8%)	192 (25.7%)	284 (25.9%)	0.90
Other prolonged/serious illness	35 (1.9%)	19 (2.5%)	16 (1.5%)	0.10
Summary Nil condition At least one condition median (range) no. of conditions	230 (12.5%) 1613 (87.5%) 3 (1–14)	98 (13.1%) 650 (86.9%) 3 (1–14)	132 (12.1%) 963 (87.9%) 2 (1–10)	0.50 0.005

Notes: Missing data present only in cancer site and SES.

Comparison between sex were performed using independent-sample t-test (for mean), chi-square test (for categorical variables), or Mann–Whitney test (for median).

Sex differences with a higher prevalence of multimorbidity were highlighted in bold for those with individual *P*-value < 0.005.

### Overall survival rates

**Figure 1:**
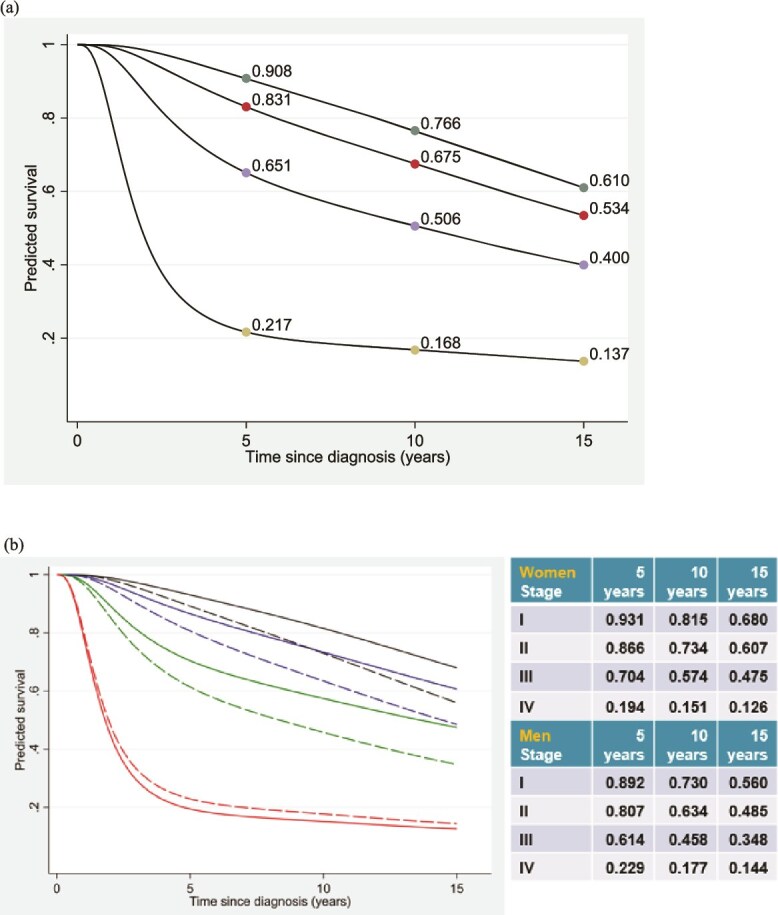
(a) Population averaged survival curves (adjusted multivariable model for death from all causes) by stage at diagnosis (from top to bottom): stages I, II, III, and IV. (b) Population averaged survival curves by sex (adjusted multivariable model for death from all causes) by stage at diagnosis (from top to bottom): stages I, II, III, and IV (women, solid lines; men, dashed lines).

The overall 5-year survival rates were 90.8%, 83.1%, 65.1%, and 21.7% for patients diagnosed with Stages I to IV cancer, respectively (Fig. 1a). The overall 15-year survival rates were, respectively, 61.0%, 53.4%, 40.0%, and 13.7%. Generally, men had significantly lower survival than women if diagnosed at Stages I–III (Fig. 1b). However, this sex difference was not observed for patients diagnosed with Stage IV.

### Risk factors of all-cause mortality

Compared to women, men had a significantly higher risk of death for patients diagnosed at Stages I–III (AHR 1.60, 1.46, and 1.35, respectively, Table 2). An interaction between sex and marital status was also observed. Marital status was not associated with survival for women. Men who were married, in a de-facto relationship (AHR 0.55) or widowed/divorced (AHR 0.61) had a decreased risk of death compared to never married men.

Men with CRC and coexisting stroke and high cholesterol (AHR 2.44) or leukaemia (AHR 4.65) had a significantly increased risk of death. However, diabetes was associated with a significantly increased risk of death (AHR 1.38) in women only.

Specific multimorbidity was associated with an increased risk of all-cause mortality for both sexes, such as cardiovascular diseases (e.g. heart attacks, AHR 1.31; other heart conditions, AHR 1.19), emphysema (AHR 1.50), osteoporosis (AHR 1.30), any previous cancer (AHR 1.42), or other prolonged/serious illness (AHR 2.09). Alternatively, patients had a reduced risk of death for those treated by surgery alone (AHR 0.47) or surgery combined with adjuvant therapy (AHR 0.50) compared to nonsurgical treatments, with private health insurance (AHR 0.85) or those living in communities with a higher SES (e.g. AHR 0.75 in the 5th quintile areas). The patient’s age and stage had time-varying effects. The AHR for age decreased and then increased after diagnosis; older patients would have worse survival compared to younger patients across time (Supplementary Fig. S1a). The AHR for men versus women decreased after diagnosis (Supplementary Fig. S1b).

### Separate risk factors of mortality due to cancer and non-cancer causes

Table 3 presents different risk factors of cause-specific mortality due to cancer and non-cancer causes. Older patients had a significantly higher risk for cancer (AHR 1.03 per year older) and non-cancer cause of death (AHR 1.14 per year older), but the effect was larger for non-cancer death. The cancer stage, treatment type, and marital status (for men only) were significant risk factors for cancer death, where sex differences were found for stage (a higher risk of cancer death for men diagnosed with Stages II (AHR 1.61) or III (AHR 1.41) and marital status (a significant risk factor for men only and that widowed/divorced men had a reduced risk of cancer death (AHR 0.38) compared to men who never married). On the other hand, socioeconomic factors (private health insurance and SES) were significant risk factors for non-cancer death, where the risk of non-cancer death was decreased for patients having private health insurance (AHR 0.77) or living in communities with a higher SES (e.g. AHR 0.55 for patients living in the 5th highest quintile areas).

The effects of multimorbidity on cancer and non-cancer death were different. Men with coexisting stroke and high cholesterol (AHR 2.22) or leukaemia (AHR 6.36) had a significantly higher risk of cancer death. Commonly to both sexes, the risk of cancer death was increased for patients with any previous cancer (AHR 1.57) or prolonged/serious illness (AHR 2.41). On the risk of non-cancer death, prognostic multimorbidity for both sexes were heart attacks (AHR 1.83), stroke (AHR 6.44), other heart conditions (AHR 1.41), emphysema (AHR 1.89), and osteoporosis (AHR 1.53). The effect of high blood pressure was significant for women only (a higher risk of non-cancer death, AHR 2.0 compared to women without high blood pressure).

**Figure 2:**
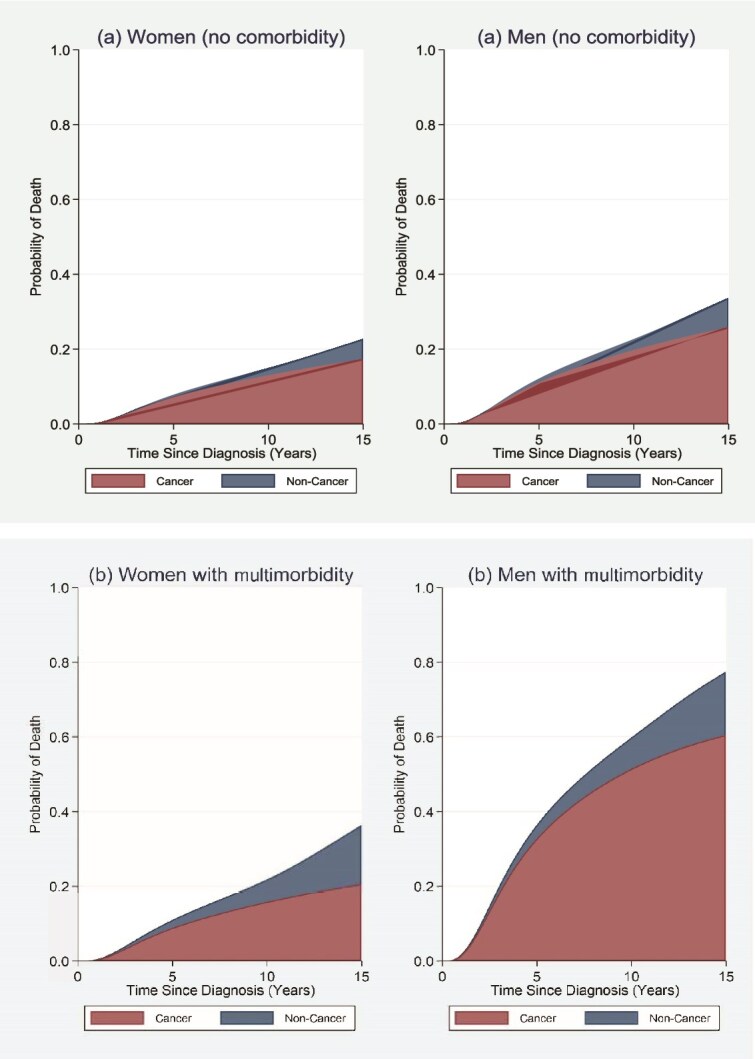
Stacked crude probabilities of death due to cancer and non-cancer causes for patients diagnosed at stage II with private health insurance, living in the 4th quintile areas, with surgical treatment only, and being married or under a de facto relationship: (a) without comorbidity, (b) with heart attacks, stroke, high cholesterol, and previous cancer.

To illustrate the sex difference in the impact of multimorbidity on cause-specific mortality, the estimated cumulative probabilities of death due to cancer and non-cancer causes for patients diagnosed with Stage II were given in Fig. 2. For women, diagnosed with cardiometabolic conditions and previous cancer increased the cumulative probability of cancer death at 15 years (from 17.3% without multimorbidity in Fig. 2a to 20.6% with multimorbidity in Fig. 2b) and non-cancer death (from 5.4% without multimorbidity to 15.8% with multimorbidity). The total cumulative probability of death at 15 years for women with multimorbidity was 36.4% (increased from 22.7% for women without multimorbidity). The impact of these comorbid NCDs was higher on men (especially, on the risk of cancer death). The cumulative probabilities of cancer and non-cancer death at 15 years were increased from 25.8% to 60.5% and from 7.9% to 17.0%, respectively. The total cumulative probability of death at 15 years for men with multimorbidity was 77.5% (increased from 33.7% for men without multimorbidity).

**Table 2:** Adjusted multivariable model for all-cause mortality (n = 1834).

Variable	Adjusted HR (95% CI)	*P*-value
Age at diagnosis^a^	1.05^b^ (1.04, 1.06)	<0.001
Stage (Men vs. women)^a^ I II III IV	1.60^b^ (1.17, 2.20) 1.46^b^ (1.15, 1.85) 1.35^b^ (1.07, 1.69) 0.80 (0.58, 1.11)	0.003 0.002 0.010 0.18
Treatment type Other treatment Surgery alone Surgery + adjuvant therapy	1.00 0.47^b^ (0.34, 0.65) 0.50^b^ (0.37, 0.69)	<0.001
Marital status (Women) Never married Married/de facto Widowed/Divorced	1.00 1.29 (0.70, 2.38) 1.55 (0.83, 2.90)	0.16
Marital status (Men) Never married Married/de facto Widowed/Divorced	1.00 0.55^b^ (0.38, 0.80) 0.61^b^ (0.40, 0.92)	0.006
Private health insurance No Yes	1.00 0.85^b^ (0.74, 0.96)	0.012
SES Quintile 1 (lowest) 2 3 4 5 (highest)	1.00 0.90 (0.73, 1.11) 0.82 (0.67, 1.00) 0.74^b^ (0.62, 0.89) 0.75^b^ (0.60, 0.95)	0.015
Multimorbidity		
Heart attacks	1.31^b^ (1.05, 1.62)	0.015
Stroke	2.24^b^ (1.12, 4.52)	0.024
Stroke × High cholesterol^c^ (Women) Stroke × High cholesterol^c^ (Men)	1.23 (0.63, 2.42) **2.44**^b^ **(1.49, 4.01)**	0.55 <0.001
Other heart condition	1.19^b^ (1.01, 1.41)	0.039
Emphysema of lungs	1.50^b^ (1.11, 2.02)	0.007
Osteoporosis	1.30^b^ (1.00, 1.68)	0.050
Leukaemia (Women) Leukaemia (Men)	0.68 (0.27, 1.72) **4.65**^b^ **(2.28, 9.49)**	0.42 <0.001
Diabetes (Women) Diabetes (Men)	**1.38** ^b^ **(1.02, 1.86)** 0.97 (0.78, 1.21)	0.039 0.78
Any previous cancer	1.42^b^ (1.21, 1.68)	<0.001
Other prolonged or serious illness	2.09^b^ (1.42, 3.09)	<0.001

Flexible parametric survival model with 4 degrees of freedom (d.f.) for the baseline spline hazard function.

AHRs were given for both sexes separately when the interaction terms regarding sex difference were significant. Sex differences in the multimorbidity effect were highlighted in bold.

HR = 1.00 indicates the reference category.

a Age and Stage effects were time dependent modelled by splines with two and one degree of freedom, respectively, the AHRs reported for Age and Stage were the main effects (the time-varying AHRs for Age and Stage were presented in Supplementary Fig. S1).

b *P*-value < 0.05.

c Co-existing of both stroke and high cholesterol.

**Table 3:** Adjusted multivariable model for mortality due to cancer and non-cancer causes (n = 1834).

Variable	Adjusted HR (95% CI)	
	Cancer death	Non-cancer death
Age at diagnosis	1.03^b^ (1.02, 1.04)	1.14^b^ (1.12, 1.17)
Stage (Men vs. women)^a^ I II III IV	1.46 (0.91, 2.35) 1.61^b^ (1.18, 2.19) 1.41^b^ (1.08, 1.82) 0.79 (0.57, 1.10)	n.s.
Treatment type Other treatment Surgery alone Surgery + adjuvant therapy	1.00 0.43^b^ (0.30, 0.62) 0.47^b^ (0.33, 0.65)	n.s.
Marital status (Women) Never married Married/de facto Widowed/Divorced	1.00 1.32 (0.65, 2.70) 1.63 (0.78, 3.40)	n.s.
Marital status (Men) Never married Married/de facto Widowed/Divorced	1.00 0.48 (0.21, 1.08) 0.38^b^ (0.16, 0.90)	n.s.
Private health insurance No Yes	n.s.	1.00 0.77^b^ (0.61, 0.97)
SES Quintile 1 (lowest) 2 3 4 5 (highest)	n.s.	1.00 0.75 (0.53, 1.06) 0.56^b^ (0.39, 0.80) 0.58^b^ (0.42, 0.79) 0.55^b^ (0.37, 0.84)
Multimorbidity		
Heart attacks	n.s.	1.83^b^ (1.31, 2.55)
High blood pressure (Women) High blood pressure (Men)	n.s.	**2.00** ^b^ **(1.36, 2.94)** 0.82 (0.61, 1.11)
Stroke	n.s.	6.44^b^ (2.62, 15.9)
Stroke × High cholesterol^c^ (Women) Stroke × High cholesterol^c^ (Men)	1.16 (0.19, 7.26) **2.22**^c^ **(1.17, 4.21)**	n.s.
Other heart condition	n.s.	1.41^b^ (1.08, 1.85)
Emphysema of lungs	n.s.	1.89^b^ (1.20, 2.96)
Osteoporosis		1.53^b^ (1.03, 2.28)
Leukaemia (Women) Leukaemia (Men)	0.51 (0.12, 2.13) **6.36**^b^ **(3.08, 13.1)**	n.s.
Any previous cancer	1.57^b^ (1.29, 1.89)	n.s.
Other prolonged or serious illness	2.41^b^ (1.58, 3.67)	n.s.

Flexible parametric survival models (4 d.f.) for the baseline hazard functions of cancer and non-cancer deaths.

n.s.: not significant (n.s. variables had a *P*-value > 0.05, their effect sizes were not reported to highlight the differences between the two causes of death).

AHRs were given for both sexes separately when the interaction terms regarding sex difference were significant. Sex differences in the multimorbidity effect were highlighted in bold.

HR = 1.00 indicates the reference category.

a Stage effects were time dependent for cancer death, modelled by splines with two degrees of freedom, the AHRs reported for Stage were the main effects (the time-varying AHRs for Stage decreased over time after diagnosis).

b *P*-value <0.05.

c Co-existing of both stroke and high cholesterol.

## Discussion

### Main finding of this study

This population-based cohort study showed men had a significantly lower overall survival than women if diagnosed at Stages I–III, whilst the survival curves were similar between sexes for CRC patients diagnosed at Stage IV. Our results showed significant sex differences in common comorbid NCDs. The impact of multimorbidity on long-term mortality was also different between men and women.

### What is already known on this topic

Cancer survival is influenced by multiple factors, including patient characteristics and diet, tumour histology, treatment, and socioeconomic differences.^5^^,^^8^^,^^17^ Our results are consistent with those of previous studies regarding the higher mortality due to older age, multimorbidity, advanced stage at diagnosis, treatment, or socioeconomic disparities.^27–29^ Whilst women have better survival than men after a diagnosis of CRC, there is limited information on associated prognostic factors for such sex difference.

### What this study adds

We found new evidence to explain sex differences in long-term mortality. Our results showed that marital status affected mortality, particularly in men, wherever married men, including widowed or divorced, had better survival compared to those who have never been married. Our findings regarding a higher prevalence of deadly conditions such as heart attacks and emphysema in men and a higher impact of prognostic conditions stroke, high cholesterol, and leukaemia in men provided additional explanations for sex difference in long-term mortality. A higher risk of postoperative mortality due to cardiovascular events arising from sepsis-mediated systemic inflammation was increasingly evidenced.^30^

Multimorbidity influences the risk of death from cancer and non-cancer causes.^5^^,^^13^^,^^31^ We found that cardiovascular diseases, emphysema, and osteoporosis were determinants of non-cancer death in both sexes, after adjustment for patient, socioeconomic/geographical, and clinical factors. Prognostic multimorbidity for cancer death was previous cancer and prolonged illness. Interestingly, stroke and high cholesterol and leukaemia increased the risk of cancer death only in men, whereas high blood pressure increased the risk of non-cancer death only in women.

This study did not find a significant impact from several typical comorbid NCDs in cancer patients. Notably, diabetes is one of the most prevalent multimorbidity amongst patients with CRC.^13^^,^^31^ Although we found diabetes was associated with worse overall survival in women (Table 2), studies are reporting that diabetes is associated with higher cancer-related deaths compared to persons without diabetes.^32^^,^^33^ But these findings are potentially confounded by the fact that cancer patients have a much higher prevalence of diabetes compared to non-cancer individuals and thus have a higher risk of cancer-related death compared to those without diabetes. Other common comorbid NCDs are depression and obesity, which are expected to have a strong impact on cancer survival due to their association with advanced stage at diagnosis, CRC progression, or a high risk of cancer or non-cancer death.^34^^,^^35^ Our results adjusted for patient- and community-level factors showed that there was no significant independent effect of depression or obesity on long-term mortality.

A reviewer suggested conducting separate analyses for new cancer cases and individuals with previous cancer. Supplementary Table S2 reported the results for all-cause mortality. Whilst sex differences in the impact of multimorbidity were similar between groups, future research on differing effects of cancer and its treatments on patient survival is warranted.

There are existing multimorbidity measures to quantify the overall burden of chronic NCDs on an individual.^2^^,^^36^ For example, the widely used Charlson Comorbidity Index (CCI) is a weighted score which considers the presence and seriousness of 19 comorbid NCDs as well as patient age (as a categorical variable).^37^ It was used for quantifying multimorbidity burden in predicting mortality of cancer patients^38–40^ and was modified for patients with cancer^41^ (the so-called cancer-specific CCI or the National Cancer Institute (NCI) combined index). Supplementary Tables S3 and S4 show the weighting for the CCI and the cancer-specific CCI (with reference to the prognostic strength determined from our study). These indexes do not consider different weighting for men and women. However, the findings of sex differences in the impact of certain comorbid NCDs on mortality would suggest the necessity of modifying these indices to account for sex differences. These are also other important public health implications, such as modifying community health cancer survivorship programmes and cancer symptom awareness campaigns,^15^ to improve the prognosis and cancer survivor’s health and quality of life.

### Limitations of this study

This study has three limitations. Firstly, we studied the effects of cancer stage and treatment when it was first diagnosed. We did not account for what happened later (e.g. if the stage changed after initial treatment). Secondly, information on multimorbidity was self-reported. Without clinical- or medication-based diagnoses, the prevalence of multimorbidity in patients with CRC could be under- or over-estimated. Thirdly, data were sourced from QCR only. Extension of the findings to different states of Australia will need further studies.

## Conclusion

Our findings can inform guidelines, cancer management strategies, survivorship programmes, and future research for effective treatment and care of women and men with CRC and multimorbidity. Efficient management requires effective coordination of managing other existing chronic NCDs as well, bearing on the different prognostic conditions identified in men and women. Targeted multilevel public health initiatives may improve the prognosis for CRC patients.

## Supplementary Material

figS1a_fdaf012

figS1b_fdaf012

figS2a_fdaf012

figS2b_fdaf012

JPH_paper_Supplementary_file_R1_clean_fdaf012

JPH_STROBE_checklist_cohort_fdaf012

## Data Availability

Data used in this study was extracted from the Colorectal Cancer and Quality of Life Study conducted by the Cancer Council Queensland. Further information is available from the corresponding author upon request.

## Notes

**Shu Kay Ng**, Professor

**Peter Baade**, Professor

**Gary Wittert**, Professor

**Alfred K. Lam**, Professor

**Ping Zhang**, Senior Research Fellow

**Saras Henderson**, Associate Professor

**Belinda Goodwin**, Associate Professor

**Joanne F. Aitken**, Professor
