# Supplementary figures and images for: Sex differences in the impact of multimorbidity on long-term mortality for patients with colorectal cancer: a population registry-based cohort study
Source: J Public Health (Oxf). 2025 Feb 5;47(2):132–43. doi: 10.1093/pubmed/fdaf012 (PMC12123309; doi:10.1093/pubmed/fdaf012)

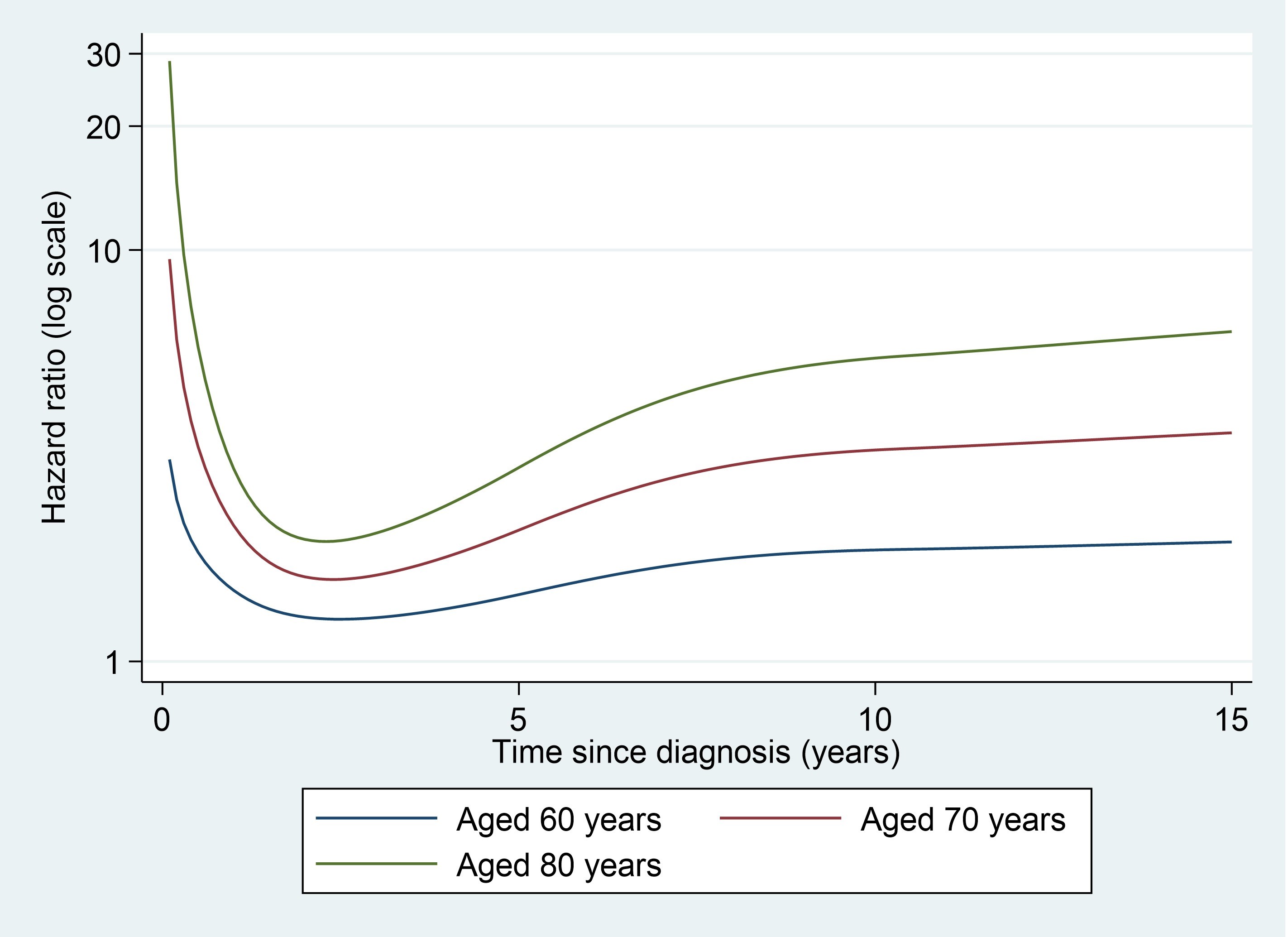

Supplement: figS1a_fdaf012 [file figs1a_fdaf012.jpeg]

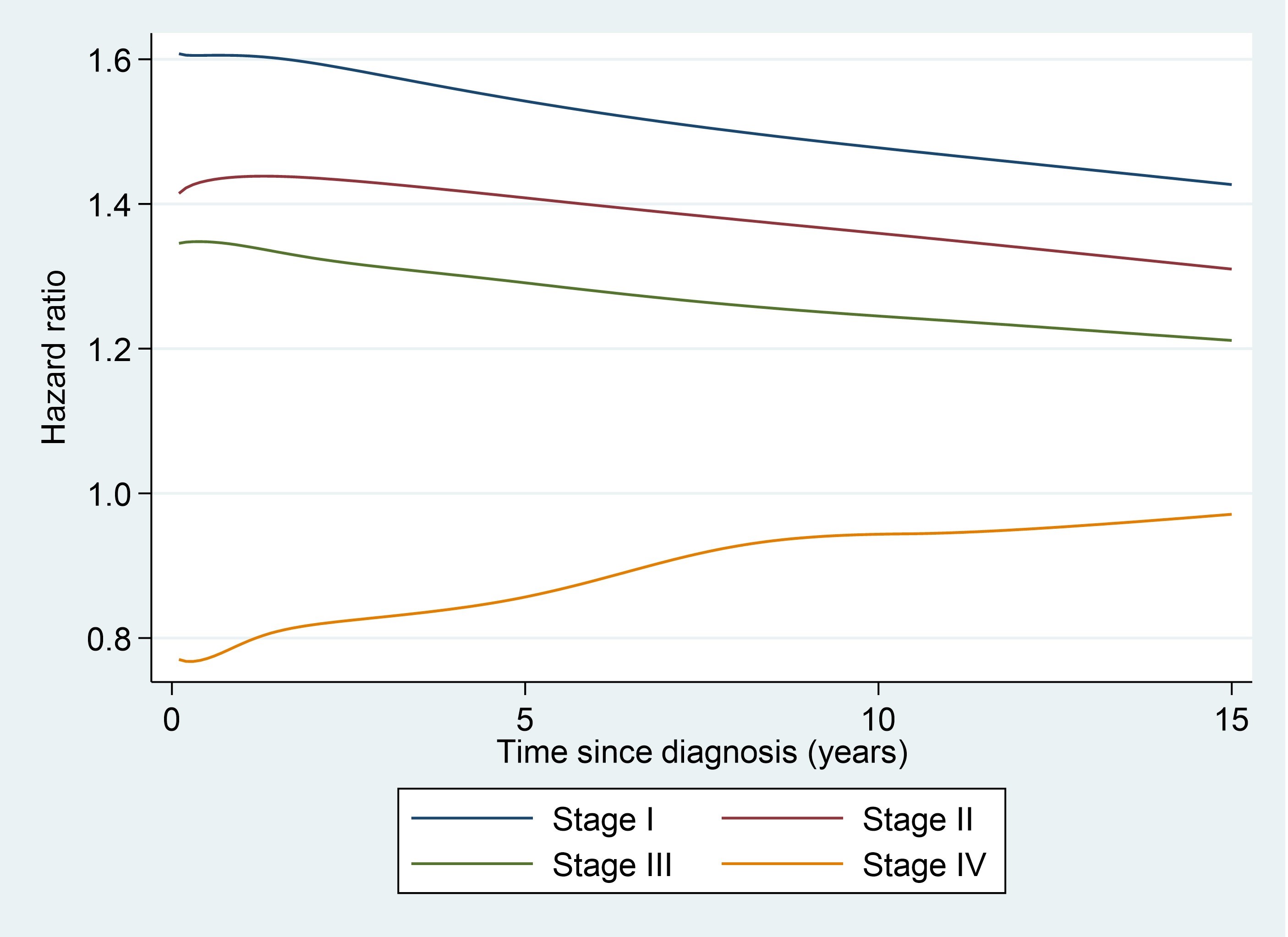

Supplement: figS1b_fdaf012 [file figs1b_fdaf012.jpeg]

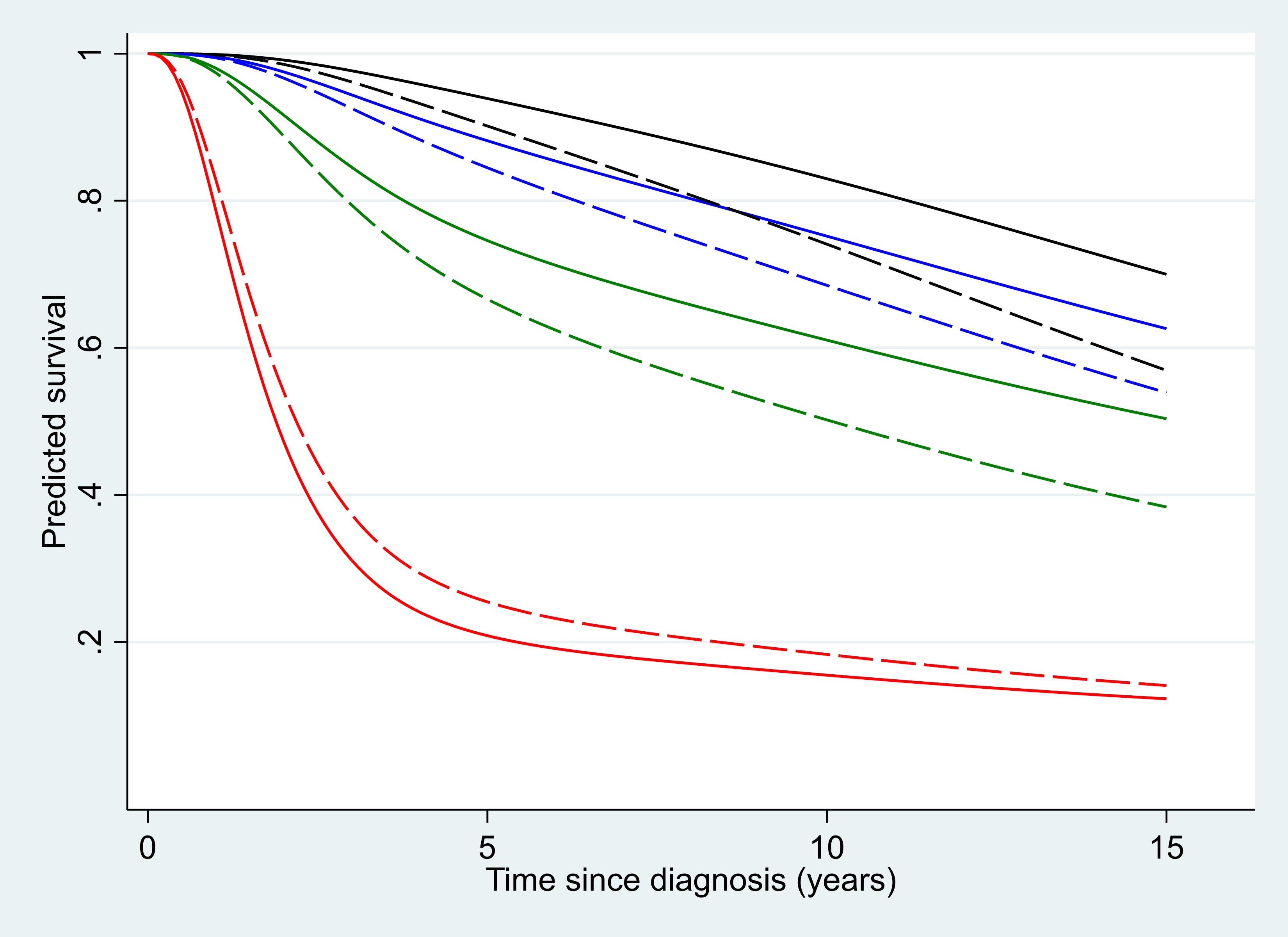

Supplement: figS2a_fdaf012 [file figs2a_fdaf012.jpeg]

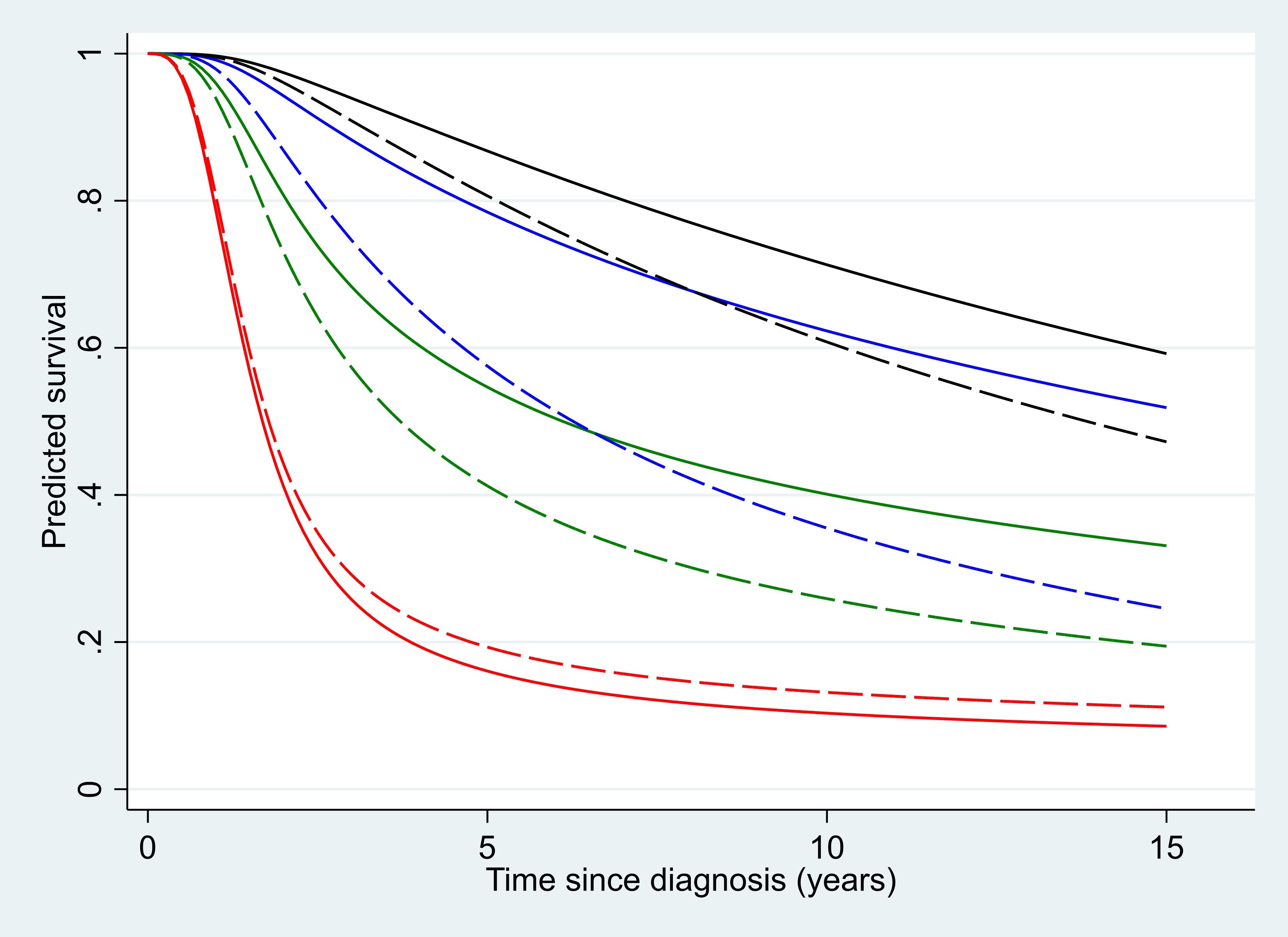

Supplement: figS2b_fdaf012 [file figs2b_fdaf012.jpeg]
