# Supplementary material for: Sex differences in the impact of multimorbidity on long-term mortality for patients with colorectal cancer: a population registry-based cohort study
Source: J Public Health (Oxf). 2025 Feb 5;47(2):132–43. doi: 10.1093/pubmed/fdaf012 (PMC12123309; doi:10.1093/pubmed/fdaf012)
Supplement: JPH_paper_Supplementary_file_R1_clean_fdaf012 [file jph_paper_supplementary_file_r1_clean_fdaf012.docx]

**Supplementary material for “Sex differences in the impact of multimorbidity on long-term mortality for patients with colorectal cancer: A population registry-based cohort study”**

Supplementary Figure S1: Adjusted hazard ratio of time-varying coefficients for all-cause mortality: (a) Age at diagnosis (with a reference of 50 years old) and (b) Sex difference (men vs women) at Stages I to IV.

Supplementary Figures S2: Population averaged survival curves by sex (adjusted multivariable model for death from all causes) by stage at diagnosis (Stage I: black, Stage II: blue, Stage III: green, Stage IV: red) (Women, solid lines; Men, dashed lines).

Supplementary Table S1: Computer-assisted telephone interview (Questions to collect information on pre-existing chronic diseases, demographics, and cancer treatment)

Supplementary Table S2: Adjusted multivariable models for all-cause mortality (New cancer cases and individuals with previous cancer)

Supplementary Table S3: Weights for the Charlson Comorbidity Index

Supplementary Table S4: The National Cancer Institute Combined Index for colorectal cancer

(a)

(b)

Supplementary Figure S1. Adjusted hazard ratio of time-varying coefficients for all-cause mortality: (a) Age at diagnosis (with a reference of 50 years old) and (b) Sex difference (men vs women) at Stages I to IV.

(a)


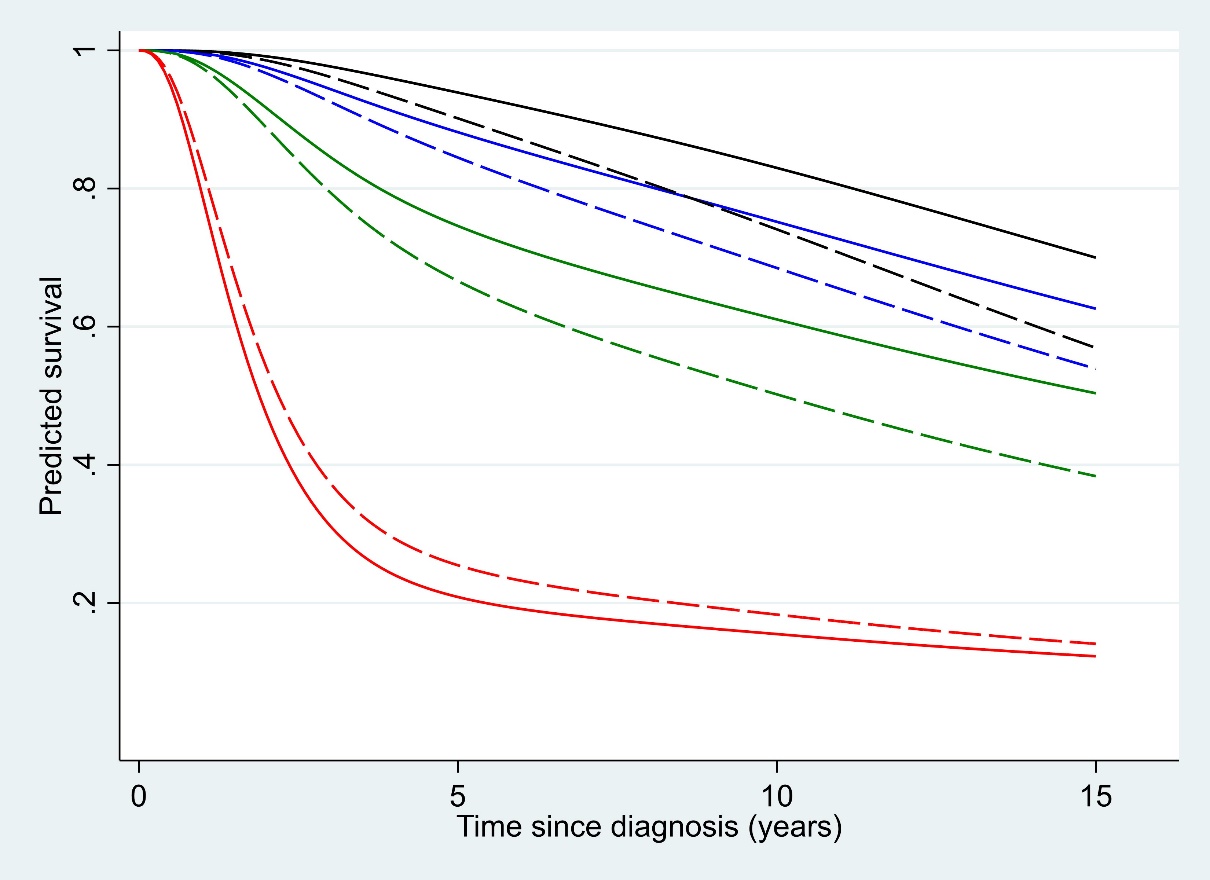


(b)


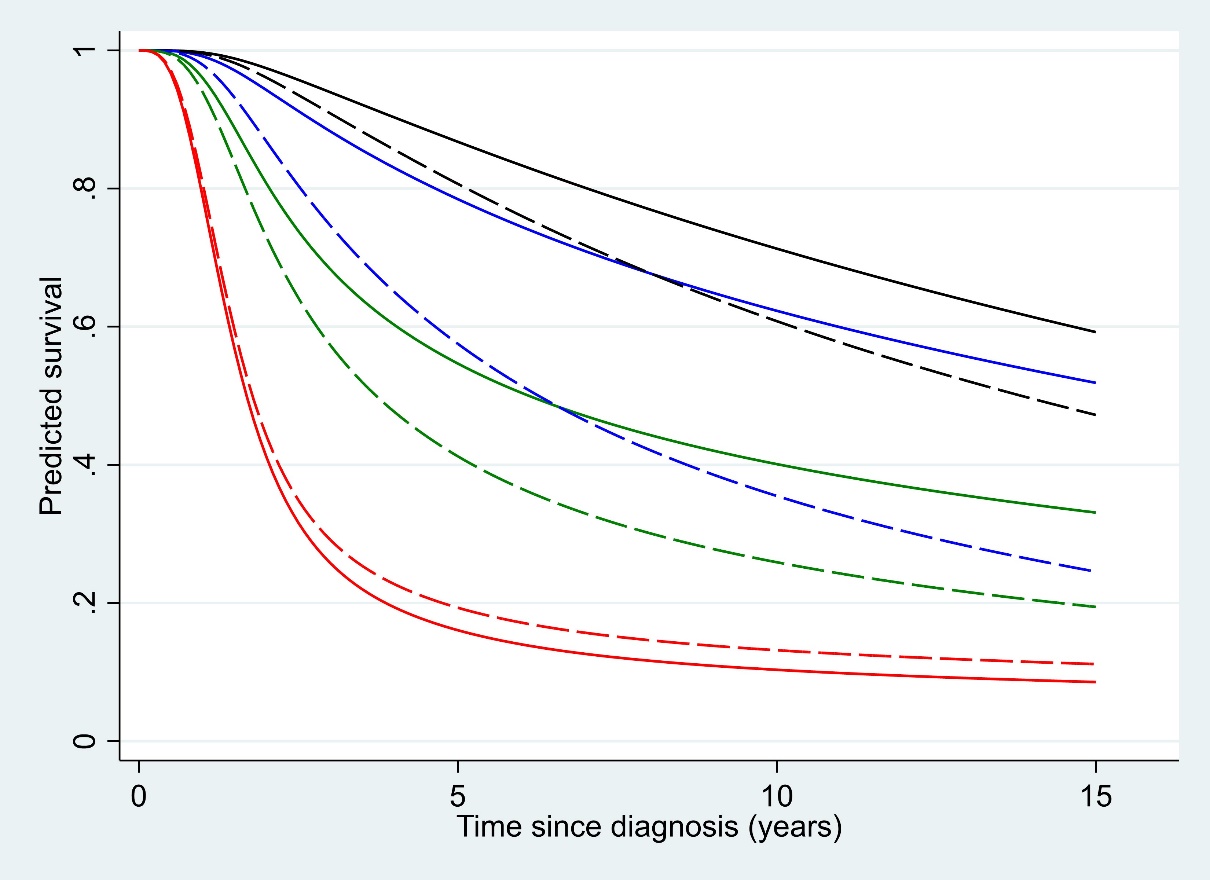


Supplementary Figures S2: Population averaged survival curves by sex (adjusted multivariable model for death from all causes) by stage at diagnosis (Stage I: black, Stage II: blue, Stage III: green, Stage IV: red) (Women, solid lines; Men, dashed lines): (a) New cancer cases, (b) Individuals with previous cancer.

Supplementary Table S1: Computer-assisted telephone interview (Questions to collect information on pre-existing chronic diseases, demographics, and cancer treatment)

| **(A) Questions to collect information on pre-existing chronic diseases** |
| --- |
| Has a doctor ever told you that you have or had any of the following conditions?  (Yes, No, or Don’t know)  Heart attack (coronary, myocardial infarction)  Angina pectoris  High blood pressure  High cholesterol  Other heart condition (Please specify)  Stroke  Diabetes  Asthma  Chronic Bronchitis  Emphysema of the lungs  Stomach or duodenal ulcer  Migraine headaches  Osteoporosis  Osteoarthritis  Rheumatoid arthritis  Depression  Leukaemia  Any previous cancer. Exclude BBCs and SCCs.  Any other prolonged or serious illness (if Yes, Which?) |
| **(B) Questions to collect information on patient’s demographics** |
| What is your date of birth? |
| What is your sex? |
| What was your weight prior to diagnosis? |
| What is your weight now? |
| What is your height? |
| Where were you born? |
| What is your current marital status?  (Never married, Now married/de facto, Widowed, Divorced/Separated) |
| What is the highest level of education you have completed?  (Less than 8 years, 8-11 years, 12 years (completed high school), Technical College, University) |
| Is (insert postal address) your correct postal address? If No, can you please tell me your correct postal address? |
| Do you have any private health insurance?  (No, Yes (ancillary cover only), Yes (basic hospital cover only), Yes (full health insurance), Department of Veterans’ Affairs (DVA) cover, Other (specify)) |
| **(C) Questions to collect information on treatment for colorectal cancer** |
| Which of the following treatments have you received for your colorectal cancer?  Surgical removal of the cancer  Chemotherapy (via drip or tablets)  Radiotherapy (radiation treatment)  Other (specify)  Awaiting treatment  No treatment – on doctor’s advice  No treatment – refused |
| With your permission, we would like to approach your doctor(s) to ask the details of your colorectal cancer and your treatment. Do you have the name and address of your treating doctors?  (GP or, if applicable, Surgeon, Medical oncologist, Radiation oncologist, or Other doctor) |

Supplementary Table S2: Adjusted multivariable models for all-cause mortality (New cancer cases and individuals with previous cancer)

| Variable | New cancer cases (n=1,482)  Adjusted HR (95% CI) | Individuals with previous cancer (n=362)  Adjusted HR (95% CI) |
| --- | --- | --- |
| Age at diagnosis† | 1.05* (1.04, 1.06) | 1.04* (1.02, 1.05) |
| Stage (Men vs. women)†  I  II  III  IV | 1.71* (1.20, 2.42)  1.30* (1.01, 1.70)  1.22 (0.94, 1.58)  0.91 (0.54, 1.52) | 1.24 (0.57, 2.73)  2.22* (1.28, 3.85)  1.81* (1.07, 3.05)  0.69 (0.44, 1.07) |
| Treatment type  Other treatment  Surgery alone  Surgery + adjuvant therapy | 1.00  0.56* (0.37, 0.84)  0.57* (0.38, 0.86) | 1.00  0.35* (0.19, 0.65)  0.34* (0.20, 0.58) |
| Marital status (Women)  Never married  Married/de facto  Widowed/Divorced | 1.00  2.23 (0.91, 5.51)  2.77* (1.11, 6.93) | 1.00  0.67 (0.28, 1.60)  0.67 (0.27, 1.65) |
| Marital status (Men)  Never married  Married/de facto  Widowed/Divorced | 1.00  0.66 (0.41, 1.07)  0.82 (0.49, 1.37) | 1.00  0.34* (0.18, 0.66)  0.30* (0.14, 0.63) |
| Private health insurance  No  Yes | 1.00  0.86 (0.74, 1.01) | 1.00  0.87 (0.67, 1.13) |
| SES Quintile  1 (lowest)  2  3  4  5 (highest) | 1.00  0.91 (0.71, 1.17)  0.75* (0.59, 0.95)  0.75* (0.60, 0.93)  0.70* (0.53, 0.92) | 1.00  0.89 (0.59, 1.34)  0.88 (0.59, 1.30)  0.64* (0.44, 0.92)  0.87 (0.56, 1.35) |
| Multimorbidity | | |
| Heart attacks | 1.60* (1.26, 2.02) | 0.71 (0.38, 1.30) |
| Stroke | 2.94* (1.28, 6.75) | 2.42 (0.54, 10.8) |
| Stroke × High cholesterol‡ (Women)  Stroke × High cholesterol‡ (Men) | 1.28 (0.62, 2.63)  2.43* (1.47, 4.02) | 0.96 (0.11, 8.20)  n.a. |
| Other heart condition | 1.06 (0.87, 1.28) | 1.76* (1.21, 2.58) |
| Emphysema of lungs | 1.63* (1.15, 2.31) | 1.79 (0.95, 3.40) |
| Osteoporosis | 1.31 (0.97, 1.76) | 1.27 (0.71, 2.26) |
| Leukaemia (Women)  Leukaemia (Men) | 0.67 (0.24, 1.90)  4.30* (2.00, 9.24) | 0.80 (0.05, 11.8)  11.8* (1.50, 93.3) |
| Diabetes (Women)  Diabetes (Men) | 1.45* (1.03, 2.05)  1.03 (0.80, 1.32) | 1.32 (0.68, 2.57)  0.97 (0.61, 1.55) |
| Other prolonged or serious illness | n.a. | 2.09* (1.40, 3.14) |

Flexible parametric survival model with 4 degrees of freedom (d.f.) for the baseline spline hazard function.

† New cancer cases: Age and Stage effects were time dependent modelled by splines with one degree of freedom;

Individuals with previous cancer: Stage effect was time dependent modelled by splines with one degree of

freedom (the AHRs reported were the main effects).

* *P* value <0.05.

‡ Co-existing of both stroke and high cholesterol.

AHRs were given for both sexes separately when the interaction terms regarding sex difference were significant. Sex differences in the multimorbidity effect were highlighted in grey.

HR = 1.00 indicates the reference category.

From Supplementary Table S2, it is observed that the effects of demographic, socioeconomic, and clinical factors were similar between new cancer cases and individuals with previous cancer (except marital status for women). Sex differences in the impact of multimorbidity were similar between groups (but statistical significance is affected by the between-group difference in sample size). While the effect of heart attacks was significant for new cancer cases, it was the effect from other heart conditions that was significant for individuals with previous cancer. These results warrant further studies on the potential differing effects of cancer and its treatment on cancer survival.

Supplementary Table S3: Weights for the Charlson Comorbidity Index

| **Condition** | Weight | Comment (reference to our study) |
| --- | --- | --- |
| AIDS | 6 | Condition not available |
| Cerebrovascular disease (CVD) | 1 | Stroke was associated with an increased risk of all-cause mortality for both sexes (AHR 2.24). **For men with comorbid stroke and high cholesterol, the risk of all-cause mortality was further increased (AHR 2.44).** |
| Chronic pulmonary disease | 1 | Chronic respiratory conditions include asthma, chronic bronchitis, and emphysema. Emphysema was associated with an increased risk of all-cause mortality for both sexes (AHR 1.50). |
| Congestive heart failure (CHF) | 1 | Condition not available |
| Connective tissue disease | 1 | Condition not available |
| Dementia | 1 | Condition not available |
| Diabetes | 1 | **Diabetes was associated with an increased risk of all-cause mortality for women only (AHR 1.38).** |
| Diabetes (with end-organ damage) | 2 |  |
| Hemiplegia | 2 | Condition not available |
| Renal disease (moderate or severe) | 2 | Condition not available |
| Leukaemia | 2 | **Leukaemia was associated with an increased risk of all-cause mortality for men only (AHR 4.65).** |
| Liver disease (mild) | 1 | Condition not available |
| Liver disease (moderate or severe) | 3 | Condition not available |
| Lymphoma | 2 | The condition may be included in “any previous cancer” below. |
| Myocardial infarct | 1 | Heart attacks were associated with an increased risk of all-cause mortality for both sexes (AHR 1.31). Other ischaemic heart related conditions include angina pectoris and “other heart condition”. Other heart condition was associated with an increased risk of all-cause mortality for both sexes (AHR 1.19). |
| Peripheral vascular disease | 1 | The condition may be included in “other heart condition” above. |
| Tumour (any) | 2 | Any previous cancer was associated with an increased risk of all-cause mortality for both sexes (AHR 1.42). Information on the stage of previous cancer was not available. |
| Tumour (solid, metastatic) | 6 |  |
| Ulcer disease | 1 | Gastric or duodenal ulcer (effect on all-cause mortality not found). |
| **Age** |  | Older patients had an increased risk of all-cause mortality for both sexes (main effect: AHR 1.05 per year older). |
| <50 years | 0 |  |
| 50-59 years | 1 |  |
| 60-69 years | 2 |  |
| 70-79 years | 3 |  |
| ≥80 years | 4 |  |

Weights were given in Charlson et al. (J Chronic Dis 1987;40:373-383).

Sex differences in effects on the risk of all-cause mortality were bolded.

AHR: Adjusted hazard ratio.

Supplementary Table S4: The National Cancer Institute Combined Index for colorectal cancer

| **Condition** | Coef. | HR | Comment (reference to our study) |
| --- | --- | --- | --- |
| Cerebrovascular disease (CVD) | 0.545 | 1.73 | Stroke was associated with an increased risk of all-cause mortality for both sexes (Coef. 0.808, AHR 2.24). **For men with comorbid stroke and high cholesterol, the risk of all-cause mortality was further increased (Coef. 0.892, AHR 2.44).** |
| Chronic pulmonary disease | 0.471 | 1.60 | Chronic respiratory conditions include asthma, chronic bronchitis, and emphysema. Emphysema was associated with an increased risk of all-cause mortality for both sexes (Coef. 0.406, AHR 1.50). |
| Congestive heart failure (CHF) | 0.836 | 2.31 | Condition not available |
| Rheumatologic disease | -0.020 | 0.98 | Rheumatoid arthritis (effect on all-cause mortality not found). |
| Dementia | 0.596 | 1.82 | Condition not available |
| Diabetes | 0.443 | 1.56 | **Diabetes was associated with an increased risk of all-cause mortality for women only (Coef. 0.322, AHR 1.38).** |
| Diabetes (with complications) | 0.302 | 1.35 |  |
| Paralysis (Hemiplegia) | 0.295 | 1.34 | Condition not available |
| Renal disease (moderate or severe) | 0.966 | 2.63 | Condition not available |
| Myocardial infarct | -0.185 | 0.83 | Heart attacks were associated with an increased risk of all-cause mortality for both sexes (Coef. 0.267, AHR 1.31). Other ischaemic heart related conditions include angina pectoris and “other heart conditions”. Other heart condition was associated with an increased risk of all-cause mortality for both sexes (Coef. 0.176, AHR 1.19). |
| Myocardial infarct (acute) | -0.021 | 0.98 |  |
| Peripheral vascular disease | 0.284 | 1.33 | The condition may be included in “other heart condition” above. |
| Ulcer disease | 0.080 | 1.08 | Gastric or duodenal ulcer (effect on all-cause mortality not found). |

Coefficients (Coef.) and hazard ratios (HRs) were given in Klabunde et al. (Ann Epidemiol 2007;17:584-590).

Sex differences in effects on the risk of all-cause mortality were bolded.

AHR: Adjusted hazard ratio.
